# Gastric Sarcoidosis Presenting As Dyspepsia

**DOI:** 10.7759/cureus.7139

**Published:** 2020-02-29

**Authors:** Khushboo Gala, Robert T Luckett, Nihar Shah

**Affiliations:** 1 Internal Medicine, University of Louisville Hospital, Louisville, USA; 2 Gastroenterology and Hepatology, University of Louisville, Louisville, USA; 3 Gastroenterology and Hepatology, Sutter Health, Berkeley, USA

**Keywords:** dyspepsia, organic dyspepsia, gastric sarcoidosis, sarcoidosis

## Abstract

The majority of cases of dyspepsia are functional, with a very rare cause of organic dyspepsia being gastric sarcoidosis. Although gastric sarcoidosis is the most common gastrointestinal manifestation of sarcoidosis, it is asymptomatic in >99% of cases. This is a case of a 48-year-old with known pulmonary sarcoidosis who was diagnosed with gastric sarcoidosis after presenting with dyspepsia.

## Introduction

Dyspepsia is one of the most common gastrointestinal problems encountered in the outpatient setting, affecting 7%-45% of the population worldwide [1]. The majority of cases of dyspepsia are functional, with less than a third of the cases having an organic cause [2]. Clinically, functional and organic dyspepsia are indistinguishable, and a high level of suspicion is required to uncover organic causes in a patient with dyspepsia. An uncommon cause of organic dyspepsia is gastric sarcoidosis, with <150 cases described in literature to date [3]. As a disease entity, sarcoidosis is a multisystem granulomatous disorder of unknown etiology, usually affecting the lungs. Gastrointestinal involvement is rare (<1%) and is mostly asymptomatic [4]. We present a case of new-onset dyspepsia secondary to gastric sarcoidosis.

## Case presentation

**Figure 1:**
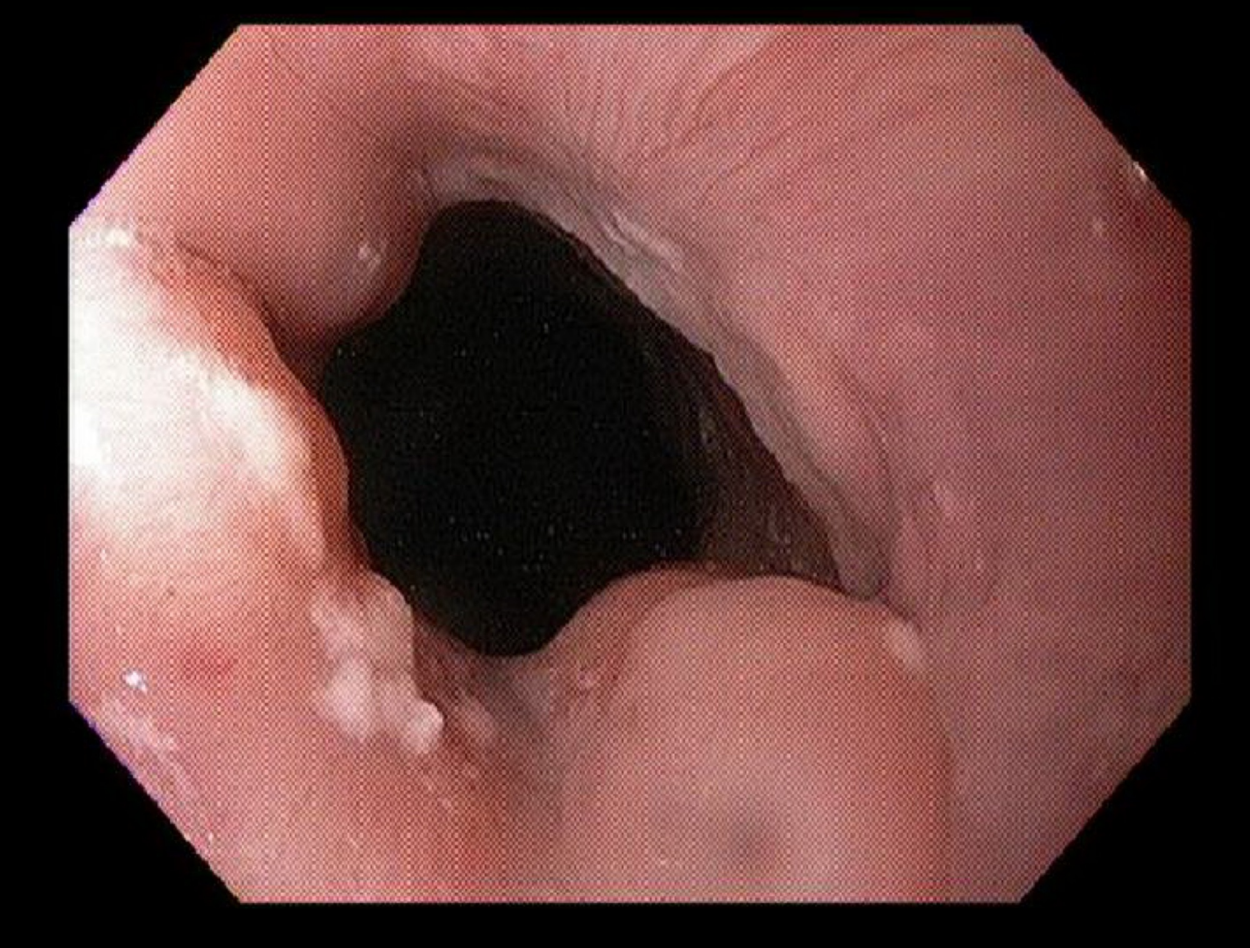
Gastroesophageal junction showing grade A erosive esophagitis

**Figure 2:**
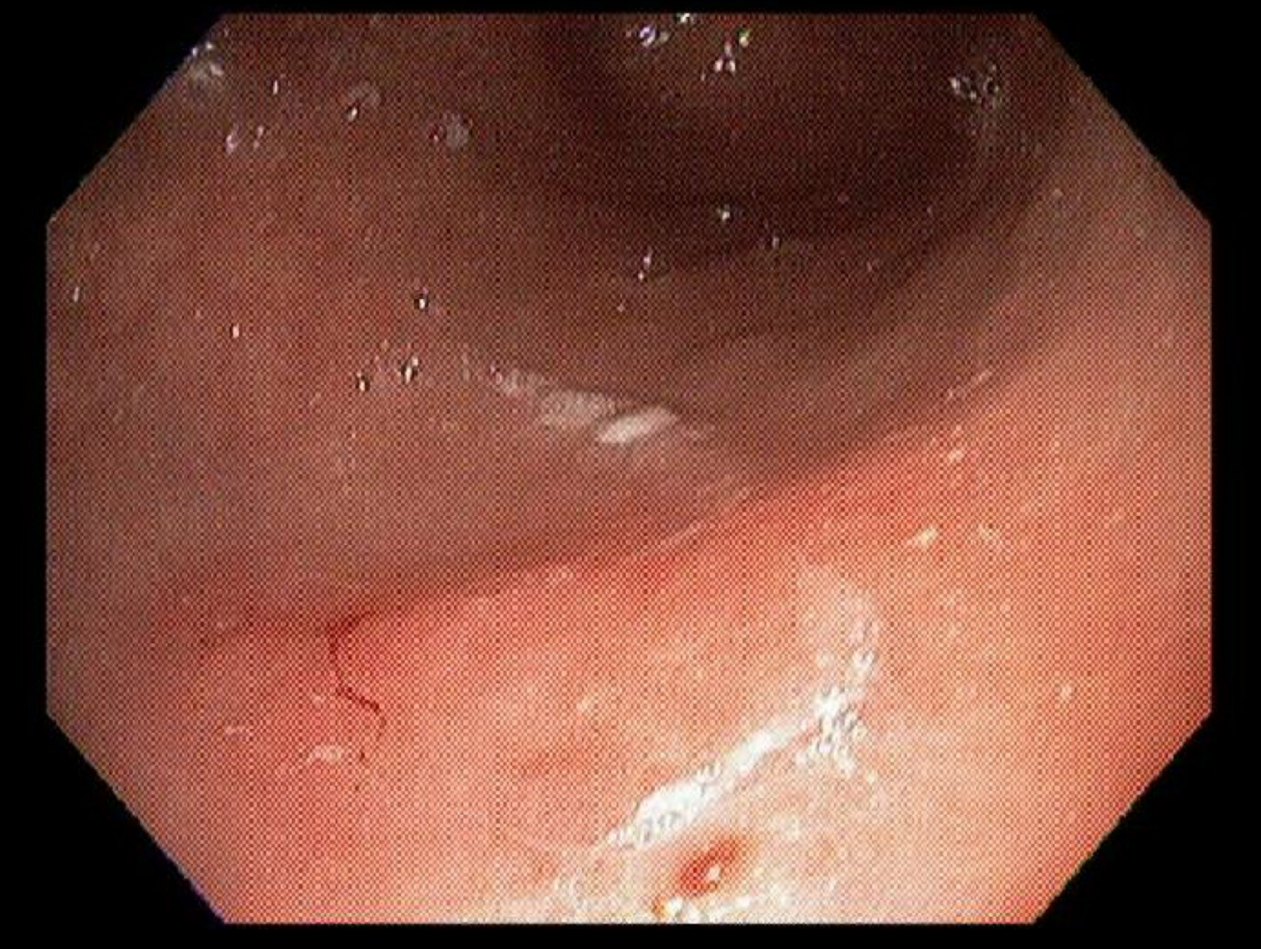
Gastric antrum showing erosive gastritis

**Figure 3:**
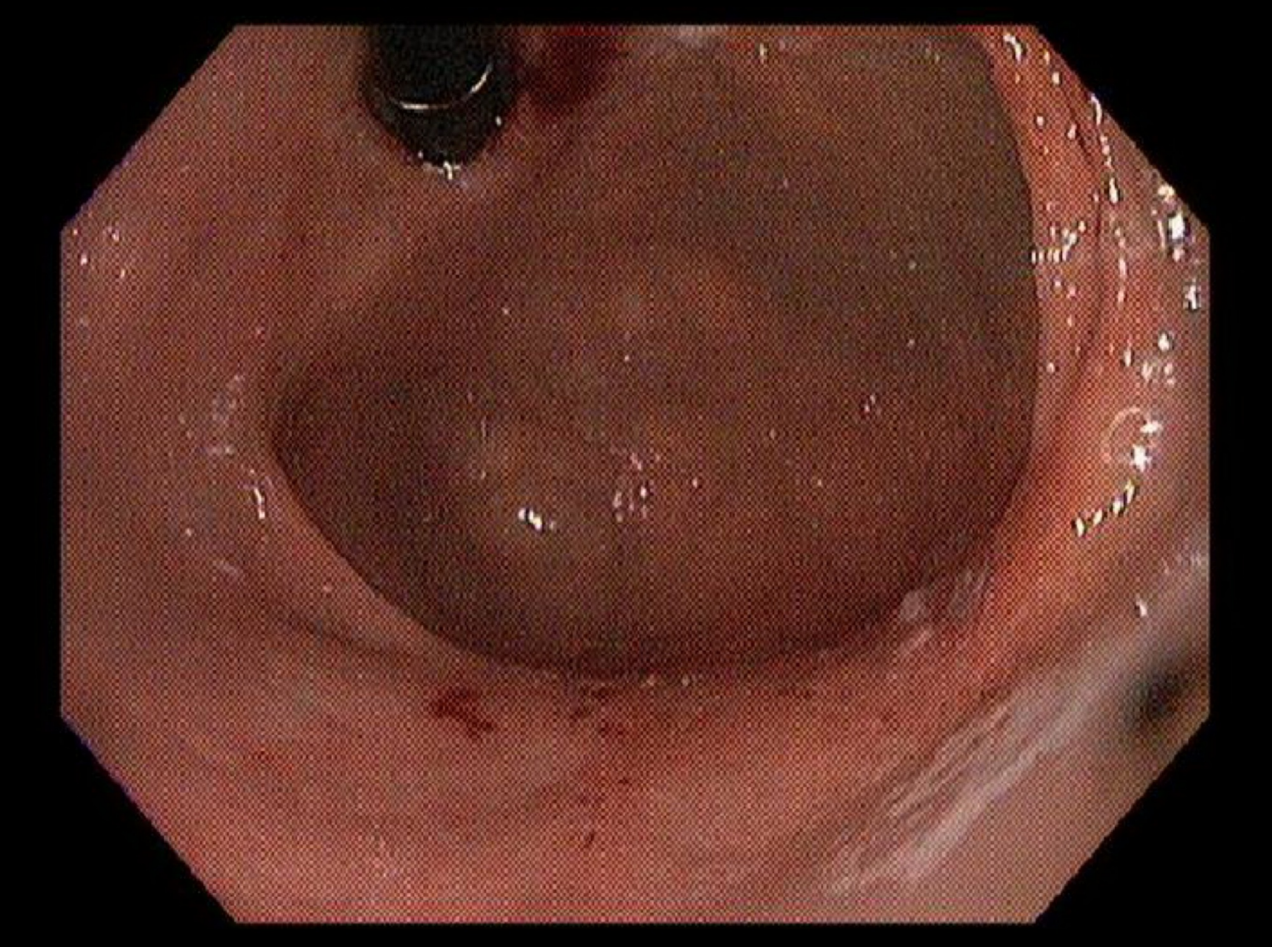
Retroflexed view of stomach showing erosive gastritis

Our patient was a 48-year-old African American female with a history of sarcoidosis, depression, and vitamin D deficiency. Her sarcoidosis was initially diagnosed by bronchoscopy and endobronchial ultrasound-guided biopsy after the patient was found to have incidental pulmonary nodules about six years prior to this presentation. She had been treated with systemic steroids and methotrexate, and had been stable off medications for one year prior to presentation. During a routine healthcare visit, she reported new onset of nausea and heartburn for one month. She was started on pantoprazole 40 mg daily, which only slightly improved her symptoms. In view of her systemic disease and advanced age, she was referred for an esophagogastroduodenoscopy, which showed Los Angeles classification grade A esophagitis of the gastroesophageal junction (Figure 1) and erosive gastritis of the antrum and body (Figures 2, 3).

**Figure 4:**
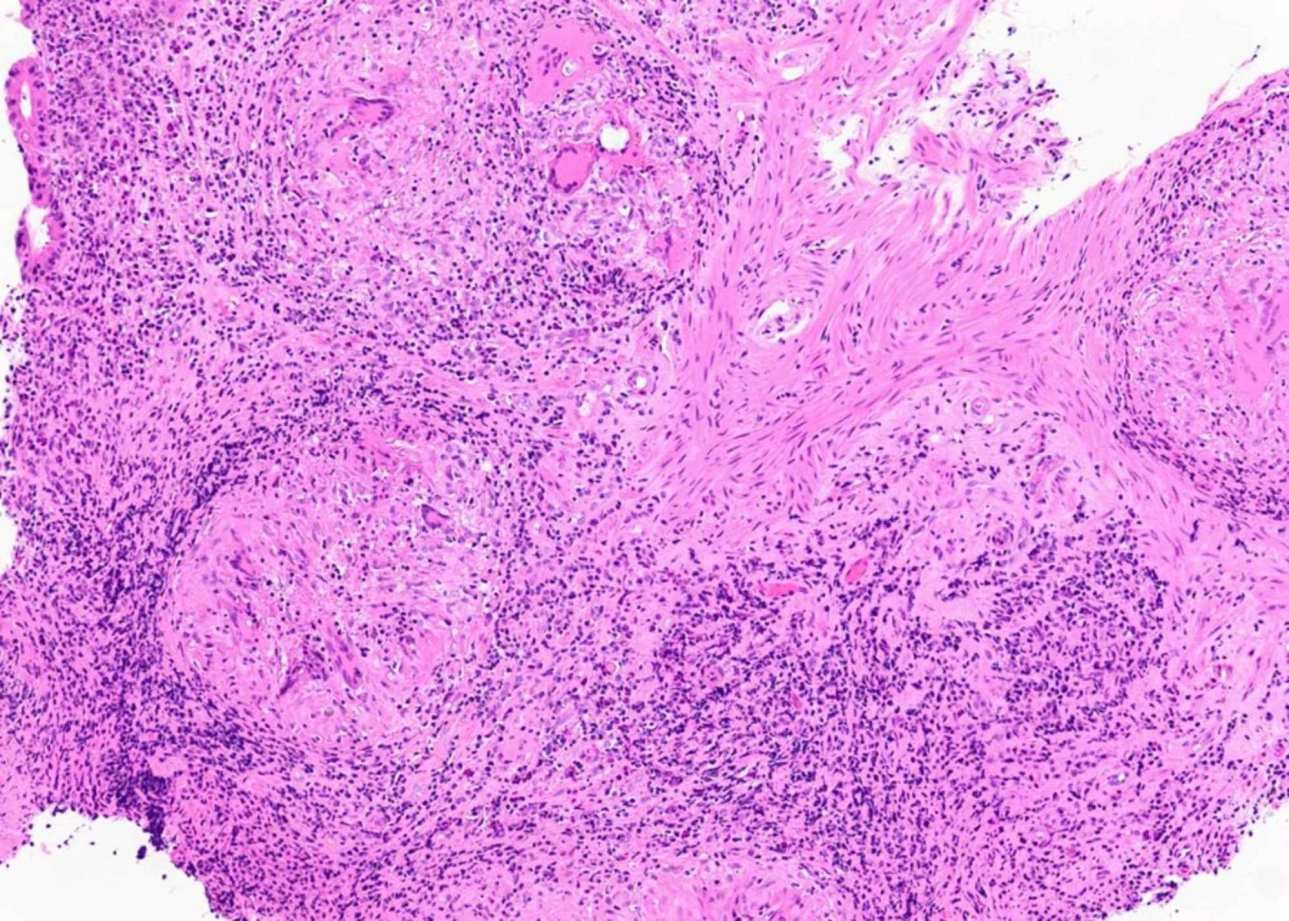
Granuloma in antral wall, H&E stain

**Figure 5:**
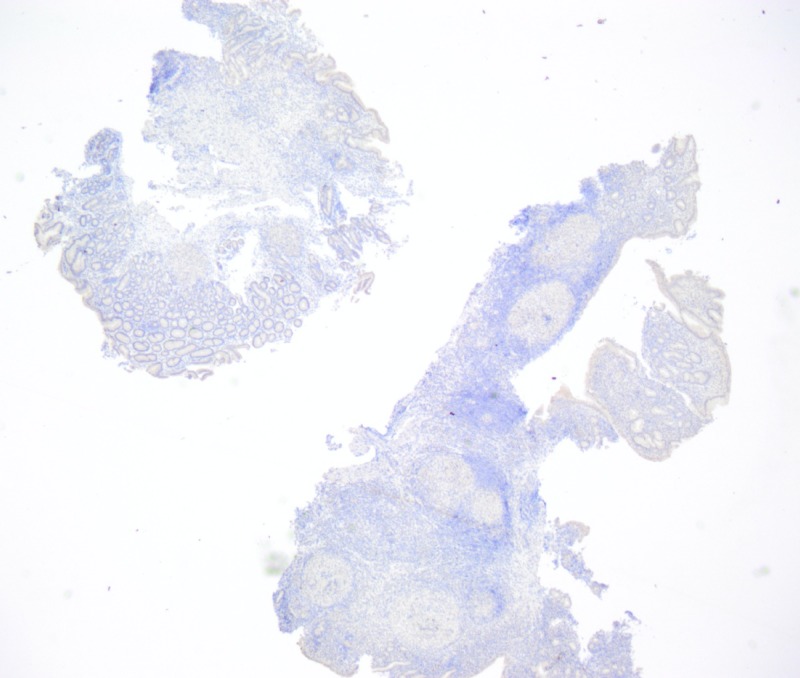
Negative Helicobacter pylori immunostain

**Figure 6:**
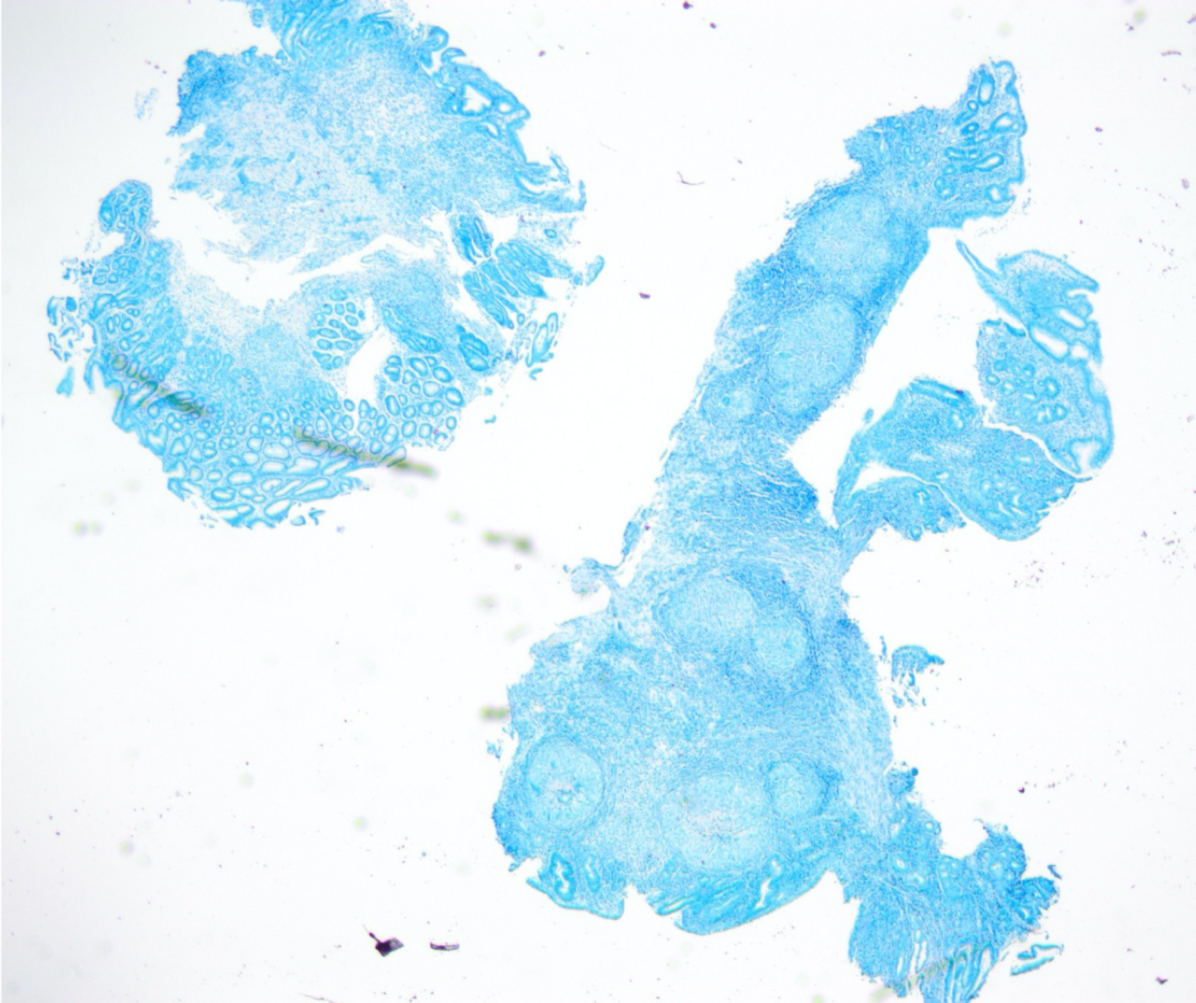
Negative acid-fast bacilli immunostain

Biopsies of gastric antrum and body showed severe active chronic gastritis with non-necrotizing granulomatous inflammation (Figure 4), with negative staining for fungi, Helicobacter pylori (Figure 5), and mycobacteria (Figure 6). These findings were consistent with a diagnosis of gastric sarcoidosis.

The patient resumed treatment with steroids and improved symptomatically, and she is now being followed outpatient.

## Discussion

Dyspepsia is a constellation of upper gastrointestinal (GI) symptoms, including the presence of upper abdominal pain or discomfort with or without other symptoms such as nausea, belching, and vomiting. Less than 30% of cases of dyspepsia are found to have an organic cause. Most cases of dyspepsia are functional, as defined by the Rome IV criteria [5]. These include having one or more of the following symptoms, and have been present for at least three months: postprandial fullness, early satiety, epigastric pain, or epigastric burning, and without an organic cause of the symptoms. The three most common organic causes of dyspepsia are peptic ulcer disease, gastroesophageal reflux (with or without esophagitis), and malignancy [6]. Among the more rare causes are motility disorders, malabsorption syndromes, ischemic disease, infectious causes including parasites, and infiltrative disorders of the stomach including Crohn’s disease and sarcoidosis.

Sarcoidosis is a multisystemic disease characterized by the histological presence of non-caseating granulomas, which most commonly manifests in the lungs. Gastrointestinal sarcoidosis is rare and usually asymptomatic, with only 0.1%-0.9% of all cases displaying symptoms [7]. The most commonly involved organ is the stomach, which is present in 10% of cases with GI involvement [8]. Most patients are asymptomatic, and studies on 18-month follow-up of such patients have confirmed that over time, there is no development of GI-related symptoms and no change in biopsy patterns [9]. Patients can also present with specific symptoms depending on the type and location of involvement, the most common of which is diffuse infiltration of the gastric antrum and body [3]. These patients present with epigastric pain, anemia, or weight loss. For patients with random gastric involvement, presenting features can vary greatly but usually include epigastric pain, nausea, or vomiting. In patients with ulcerative lesions, upper gastrointestinal bleeds are common. Patients with large, polypoid lesions can present with gastric outlet obstruction.

Regarding diagnostic approach to GI sarcoidosis, a high degree of suspicion is required as the diagnosis is made histologically with upper endoscopy and biopsy. The endoscopic appearance varies depending on the type of involvement, but can range from normal appearing mucosa to erosive, friable mucosa with nodularity and prominent folds, ulceration, or polyps. A deep mucosal or full thickness biopsy is recommended, as superficial biopsies may be non-specific. Tissue biopsy typically demonstrates the characteristic histologic findings of sarcoidosis: non-caseating granulomatous lesions with multinucleated giant cells. It is imperative to rule out infections and other etiologies of granulomas with appropriate staining. Management consists of immunosuppressive and symptomatic therapy in patients with symptoms; asymptomatic patients are usually not treated [10]. The mainstay of treatment is glucocorticoids, with the preferred agent being prednisone 0.5 mg/kg per day with a gradual taper to a maintenance dose of 10 to 15 mg daily. The duration of treatment is guided by clinical response, but typically spans a duration of at least 6-12 months. There is limited data on second-line therapies including hydroxychloroquine, azathioprine, or methotrexate. Surgery should be considered when there is severe gastric lumen narrowing or obstruction. 

Patients with dyspepsia can have significantly decreased quality of life, although their life expectancy is normal. A large number of cases of dyspepsia remain uninvestigated, with their overall pooled prevalence estimated at 21% [2]. Studies have shown that symptoms of dyspepsia do not reliably distinguish between organic and functional forms of the disease; hence, it is a clinical challenge to distinguish between the two [11]. Although rare, sarcoidosis is the second most common cause of granulomatous gastritis in developed countries and should be considered in the differential diagnosis when investigating patients with dyspepsia [12].

This article has been presented as an abstract (https://insights.ovid.com/crossref?an=00000434-201910001-02739) [13].

## Conclusions

A rare organic cause of dyspepsia is gastric sarcoidosis, which can have a variety of presentations and endoscopic appearances. It can be diagnosed by its characteristic biopsy appearance, and treated with systemic steroids when symptomatic. Our case highlights a rare and likely underdiagnosed cause of dyspepsia, the importance of maintaining a broad differential diagnosis, and appropriate use of upper endoscopy for evaluation of patients with new-onset dyspepsia.
